# Cytosolic THUMPD1 promotes breast cancer cells invasion and metastasis via the AKT-GSK3-Snail pathway

**DOI:** 10.18632/oncotarget.14528

**Published:** 2017-01-05

**Authors:** Xiupeng Zhang, Guiyang Jiang, Mingfang Sun, Haijing Zhou, Yuan Miao, Mengyuan Liang, Enhua Wang, Yong Zhang

**Affiliations:** ^1^ Department of Pathology, First Affiliated Hospital and College of Basic Medical Sciences, China Medical University, Shenyang, China; ^2^ Department of Pathology, Cancer Hospital of China Medical University, Shenyang, China

**Keywords:** THUMPD1, breast cancer, AKT signaling, snail, GSK3β

## Abstract

Human THUMP domain-containing protein 1 (THUMPD1) is a specific adaptor protein that modulates tRNA acetylation through interaction with NAT10. Immunohistochemical analysis of 146 breast cancer specimens (82 triple-negative and 64 non-triple-negative cases) indicated THUMPD1 expression is higher in breast cancer tissues (60.9%, 89/146) than normal breast tissues (28.3%, 15/53; *p* < 0.001). Overall and cytosolic, but not nuclear, THUMPD1 expression in breast cancer correlated with advanced TNM stage (*p* = 0.003 and *p* < 0.001, respectively), lymph node metastasis (*p* = 0.001 and *p* < 0.001, respectively), and poor patient prognosis (*p* = 0.001 and *p* < 0.001, respectively). THUMPD1 interacted and co-localized with YAP, but did not affect Hippo pathway activity. THUMPD1 overexpression enhanced breast cancer cells invasion and migration *in vivo* and *in vitro*, possibly through activation of AKT, GSK3β and Snail, and inhibition of E-cadherin. Treatment with the AKT inhibitor, LY294002, reduced the effects of THUMPD1 overexpression in breast cancer cells. These results indicate that THUMPD1 promotes breast cancer cells invasion and migration via the AKT-GSK3β-Snail pathway.

## INTRODUCTION

Breast cancer is the leading cause of cancer death in women [1, 2]. Despite advances in tumor diagnostic and therapeutic strategies, patient outcomes are still poor due in part to cancer cell metastasis and heterogeneity [3, 4]. Novel biomarkers are needed to more accurately identify high-risk patients and to predict disease prognosis [5]. Genes abnormally expressed during tumor progression and metastasis may be used as markers to provide prognostic information beyond standard clinical assessment [6, 7].

Human THUMP domain-containing protein 1 (THUMPD1) is a specific adaptor protein that interacts with NAT10, a human acetyltransferase involved in histone and microtubule modification, and thus modulates tRNA acetylation [8]. Havugimana, *et al*. [9] suggested that THUMPD1 interacted with Yes-associated protein (YAP), a major transcriptional co-activator of the Hippo pathway, which plays crucial roles in tumor proliferation and invasion [10–13]. Our preliminary experiments showed that THUMPD1 expression was elevated in breast carcinoma samples as compared to normal breast tissues. However, THUMPD1 downstream signaling pathways in human breast cancer are as yet largely unknown. We hypothesized that THUMPD1 may promote malignant transformation through its interaction with YAP.

To test this hypothesis, we examined THUMPD1 expression and localization in breast cancer tissues and investigated associations between THUMPD1 subcellular localization and patient clinicopathological factors. We also addressed the effects of THUMPD1 on cancer cell migration and invasion, and analyzed potential downstream signaling pathways.

## RESULTS

### THUMPD1 expression and subcellular localization in breast carcinoma

**Figure 1:**
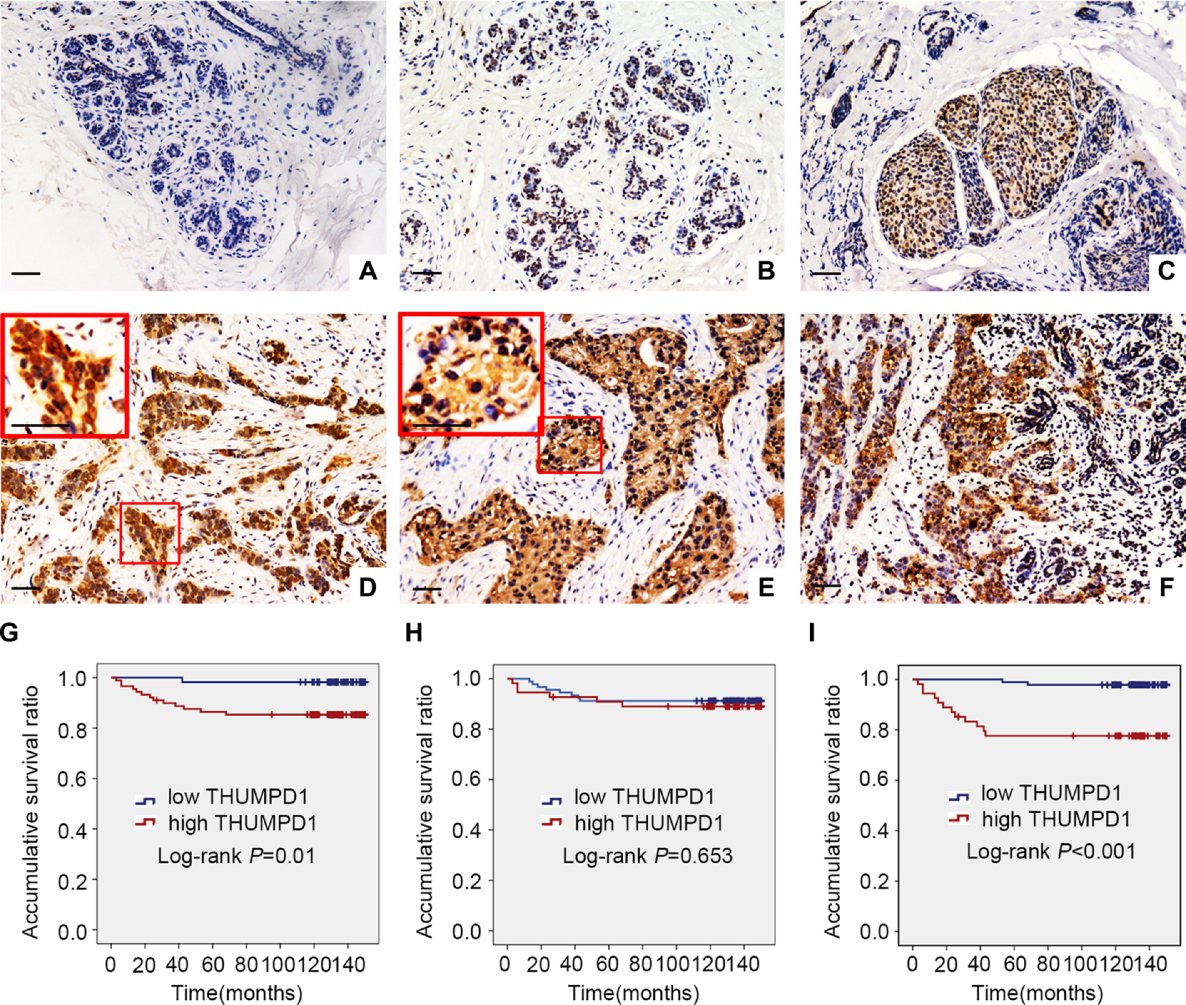
THUMPD1 expression and subcellular localization in breast tumors, and association with patient survival THUMPD1 subcellular localization as shown by immunohistochemistry. In normal breast ductal cells, THUMPD1 expression was either absent (**A**) or weak (**B**). In carcinoma in situ (**C**) and IDC cells (**D**), THUMPD1 was observed in the nucleus and cytoplasm at moderate and high levels, respectively. In some IDC specimens, THUMPD1 was exclusively localized in the cytoplasm (**E**). THUMPD1 cytosolic and nuclear expression was higher in IDC (**F**) than in normal breast ductal cells. Magnification, ×200 and ×400. Kaplan-Meier analysis demonstrated that patient overall survival negatively correlated with overall (**G**) and cytosolic (**H**), but not nuclear (**I**), THUMPD1 expression.

We performed immunohistochemistry to assess THUMPD1 expression in 146 breast cancer and 53 paired noncancerous specimens. THUMPD1 presented negative or weak nuclear expression in normal breast tissues (Figure 1A–1B), moderate nuclear expression in carcinoma *in situ* samples (Figure 1C), and strong nuclear expression in IDCs (Figure 1D), which also showed positive THUMPD1 cytosolic expression (Figure 1E). Positive THUMPD1 expression frequency was higher in IDCs (60.9%, 89/146) than in normal breast ductal epithelium (28.3%, 15/53, *p* < 0.001; Figure 1F). Cytosolic and nuclear THUMPD1 expression frequency were 37% and 37.7%, respectively, in breast cancers, and 24.5% (*p* = 0.093) and 5.6% (*p* < 0.001), respectively, in normal breast tissues. Statistical analyses revealed that overall and cytosolic THUMPD1 expression correlated with high TNM stage (*p* = 0.003 and *p* < 0.001, respectively) and lymph node metastasis (*p* = 0.001 and *p* < 0.001, respectively). However, nuclear THUMPD1 expression showed no obvious correlation with clinicopathological factors (Table 1). Kaplan-Meier analysis indicated reduced patient survival in tumors positive for overall and cytosolic THUMPD1 expression (131.86 ± 4.71 months and 121.35 ± 7.33 months, respectively) as compared with THUMPD1-negative patients (148.11 ± 1.88 months, *p* = 0.001, and 148.05 ± 1.37 months, *p* < 0.001, respectively; Figure 1G–1H). Survival of patients with and without nuclear THUMPD1 was similar (136.50 ± 5.32 months versus 139.26 ± 3.64 months, respectively, *p* = 0.653; Figure 1I). However, subsequent cox univariate (UA) and multivariate (MA) analyses revealed that cytosolic overexpression of THUMPD1 could not be considered an independent prognostic factor in breast cancer (*p* = 0.001 for UA, and *p* = 0.146 for MA, Table 2).

**Table 1:** Correlation of THUMPD1 overexpression with clinicopathological features in 146 cases breast cancer

Clinicopathological factors	The overall expression				Cytosolic expression				Nuclear expression			
	*N*	positive	negative	*p*	*N*	positive	negative	*p*	*N*	positive	negative	*p*
Age												
< 52	75	48	27	0.499	75	29	46	0.733	75	27	48	0.734
≥ 52	71	41	30		71	25	46		71	28	43	
TNM classification												
I+II	122	68	54	0.003	122	36	86	< 0.001	122	44	78	0.368
III	24	21	3		24	18	6		24	11	13	
Lymph node metastasis												
positive	30	26	4	0.001	30	20	10	< 0.001	30	16	14	0.058
negative	116	63	53		116	34	82		116	39	77	
Triple-negative(ER,PR,Her-2)												
positive	82	54	28	0.177	82	20	44	0.23	82	31	51	1
negative	64	35	29		64	34	48		64	24	40	

**Table 2:** Summary of Cox univariate and multivariate regression analysis of the association between clinicopathological features and overall survival in 146 cases of breast cancer

Clinicopathological feature	Hazard ratio (95% CI)	*P*
**Univariate analysis**		
Age	0.582 (0.195–1.728)	0.582
TNM classification	96.688 (12.594–742.282)	< 0.001
Lymph node metastasis	66.491 (8.679–509.418)	< 0.001
Triple–negative	1.023 (0.355–2.948)	0.967
Nuclear expression	1.274 (0.442–3.671)	0.654
Cytosolic expression	11.771 (2.632–52.635)	0.001
The overall expression	8.984 (1.175–68.686)	0.034
**Multivariate analysis**		
TNM classification	16.978 (1.485–194.07)	0.023
Lymph node metastasis	7.408 (0.649–84.579)	0.107
Cytosolic expression	4.531 (0.59–34.793)	0.146
The overall expression	0.821 (0.052–13.033)	0.889

**Figure 2:**
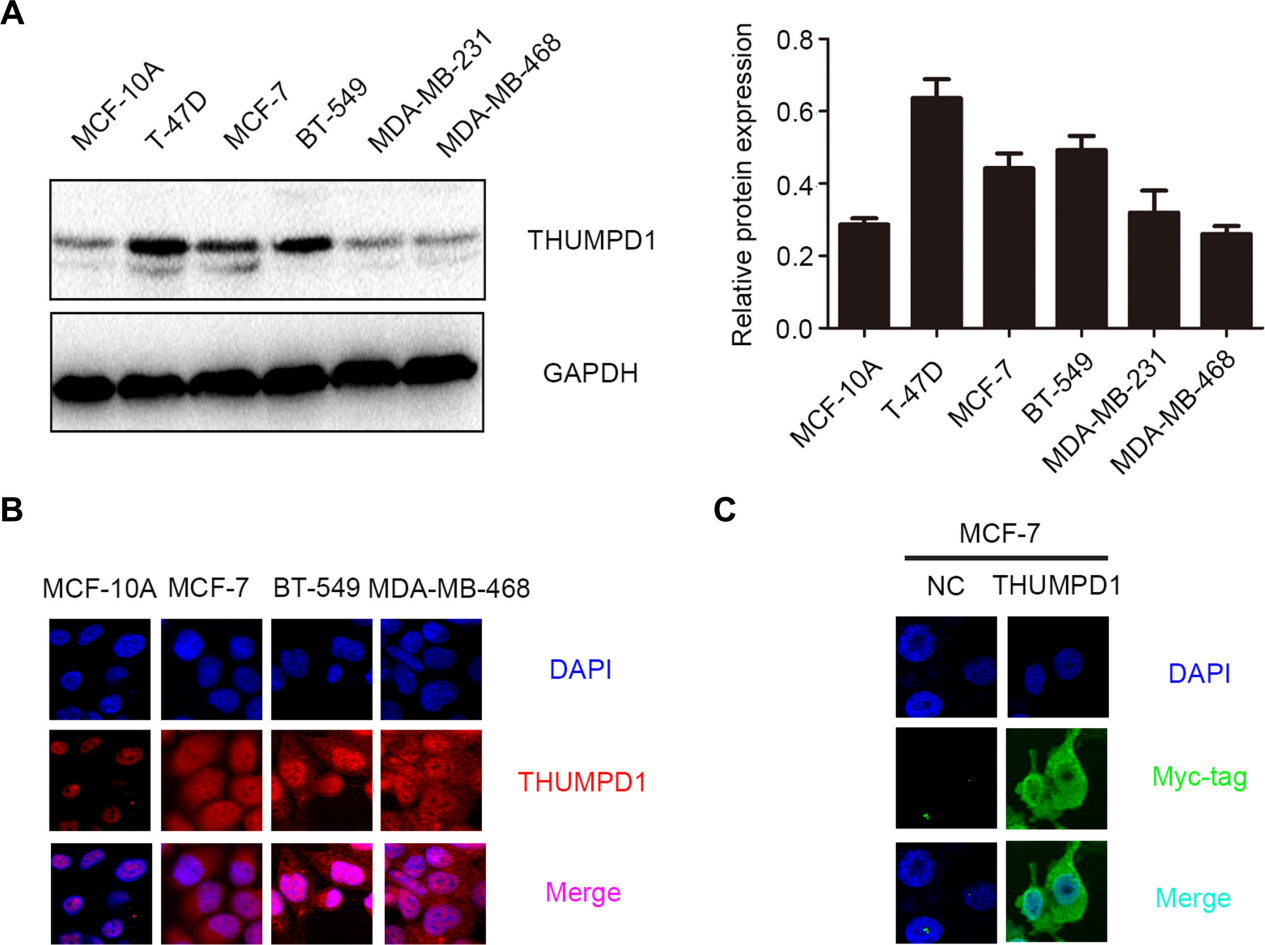
THUMPD1 expression and subcellular localization in breast cancer cell lines THUMPD1 expression was higher in most tested breast cancer cell lines compared to normal breast cells (MCF-10A), but was lower in MDA-MB-231 and MDA-MB-468 cells (**A**). Immunofluorescence analyses indicated that THUMPD1 was mainly localized in MCF-10A cell nuclei, and in both nuclei and cytoplasm of breast cancer cells (**B**). Immunofluorescence analysis of THUMPD1-myc expression in MCF-7 cells using a Myc-tag antibody (**C**). Overexpressed THUMPD1 was predominantly localized in the cytoplasm. No positive signal was detected in untransfected cells. Magnification, ×600.

We then assessed THUMPD1 expression and subcellular localization in cell lines through western blotting and immunofluorescence. All breast cancer cell lines were positive for THUMPD1 expression, whereas the normal breast ductal cell line, MCF-10A, showed only weak expression (Figure 2A). Breast cancer cells exhibited both cytosolic and nuclear THUMPD1 expression, with only nuclear expression in MCF-10A cells (Figure 2B). In MCF-7 cells transfected with the THUMPD1-myc expression plasmid, exogenous THUMPD1 was largely localized in the cytoplasm (Figure 2C).

### Interaction between THUMPD1 and YAP

**Figure 3:**
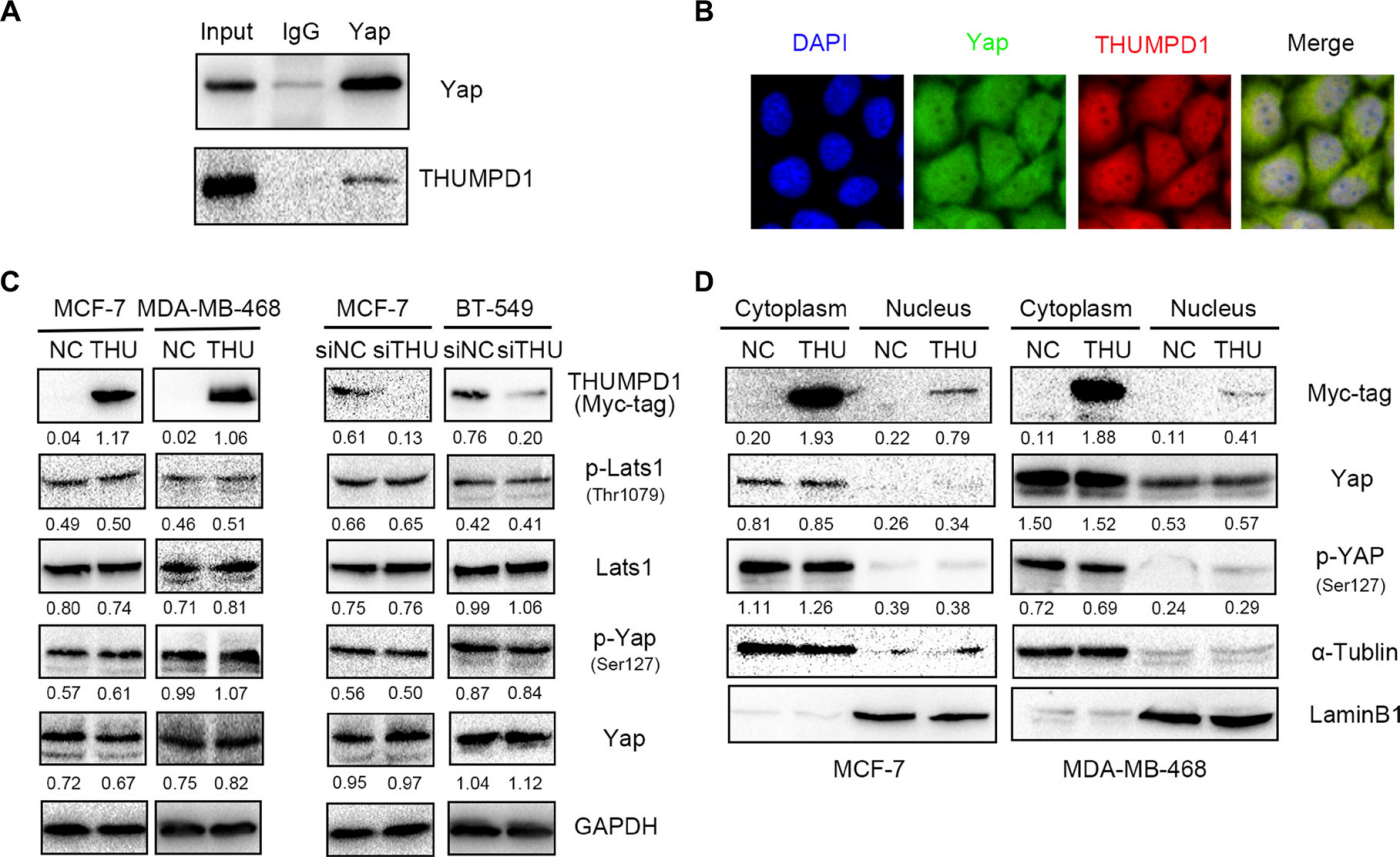
THUMPD1 interaction with YAP Co-immunoprecipitation performed with a YAP antibody demonstrated that YAP bound THUMPD1 (**A**). Immunofluorescence analyses showed that THUMPD1 co-localized with YAP in the cytoplasm and nucleus (**B**). Magnification, ×600. Western blotting analysis of THUMPD1-overexpressing MCF-7 and MDA-MD-468 cells, and THUMPD1-deficient MCF-7 and BT-549 cells, revealed that YAP, p-YAP, LATS, and p-LATS levels were unaffected by THUMPD1 (**C**). THUMPD1 overexpression did not change YAP or p-YAP levels in MCF-7 and MDA-MD-468 cell cytoplasm or nuclei (**D**).

Immunoprecipitation and immunofluorescence were performed using THUMPD1-overexpressing MCF-7 cells to investigate the interaction between THUMPD1 and YAP. Exogenous THUMPD1 interacted directly with YAP (Figure 3A), and expression was co-localized in both the cytoplasm and nucleus (Figure 3B).

We assessed YAP phosphorylation status and distribution in THUMPD1-overexpressing MCF-7 and MDA-MB-468 cells, and in THUMPD1-deficient MCF-7 and BT-549 cells. Neither overexpression nor depletion of THUMPD1 affected levels of YAP, p-YAP, or the upstream regulator, LATS and p-LATS1 (Figure 3C). YAP and p-YAP subcellular distributions (Figure 3D) were also unchanged. Therefore, THUMPD1, despite its interaction with YAP, may not influence Hippo signaling.

### THUMPD1 enhanced breast cancer cells invasion and migration

**Figure 4:**
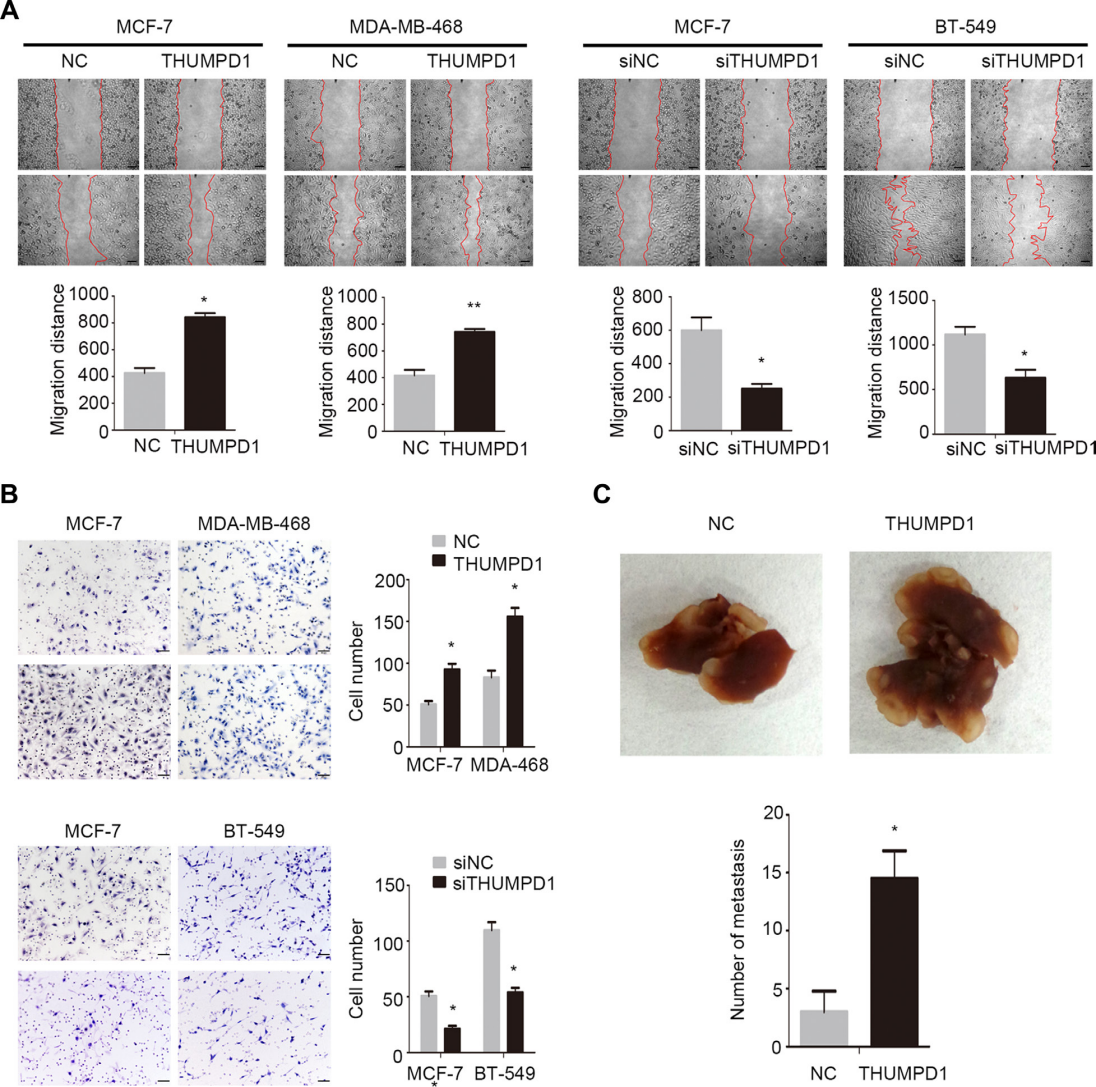
THUMPD1 enhanced breast cancer cell invasion and migration in vivo and in vitro Cell migration (**A**) Cell invasion (**B**) THUMPD1 overexpression in MCF-7 and MDA-MB-468 cells enhanced migration and invasion, while THUMPD1 knockdown in MCF-7 and BT-549 cells inhibited migration and invasion. Mice injected with THUMPD1-overexpressing MCF-7 cells developed more pulmonary metastases than controls (**C**) *p < 0.05.

We then addressed THUMPD1 effects on tumor cell invasion and migration by overexpressing THUMPD1 in MCF-7 and MDA-MB-468 cells or depleting THUMPD1 in MCF-7 and BT-549 cells. In MCF-7 and MDA-MB-468 cells, THUMPD1 overexpression enhanced migration (*p* = 0.014 and *p* = 0.008, respectively; Figure 4A) and invasion (*p* = 0.048 and *p* = 0.014, respectively; Figure 4B). In MCF-7 and BT-549 cells, THUMPD1 depletion inhibited migration (*p* = 0.020 and *p* = 0.032, respectively; Figure 4A) and invasion (*p* = 0.033 and *p* = 0.004, respectively, Figure 4B). In nude mice intravenously injected with THUMPD1-overexpressing MCF-7 cells, lung metastasis incidence was increased compared with controls (4/4 versus 2/4 in control), as were total numbers of lung metastatic nodules (*p* = 0.015; Figure 4C).

### THUMPD1 downregulated E-cadherin via AKT–GSK3β–Snail signaling

**Figure 5:**
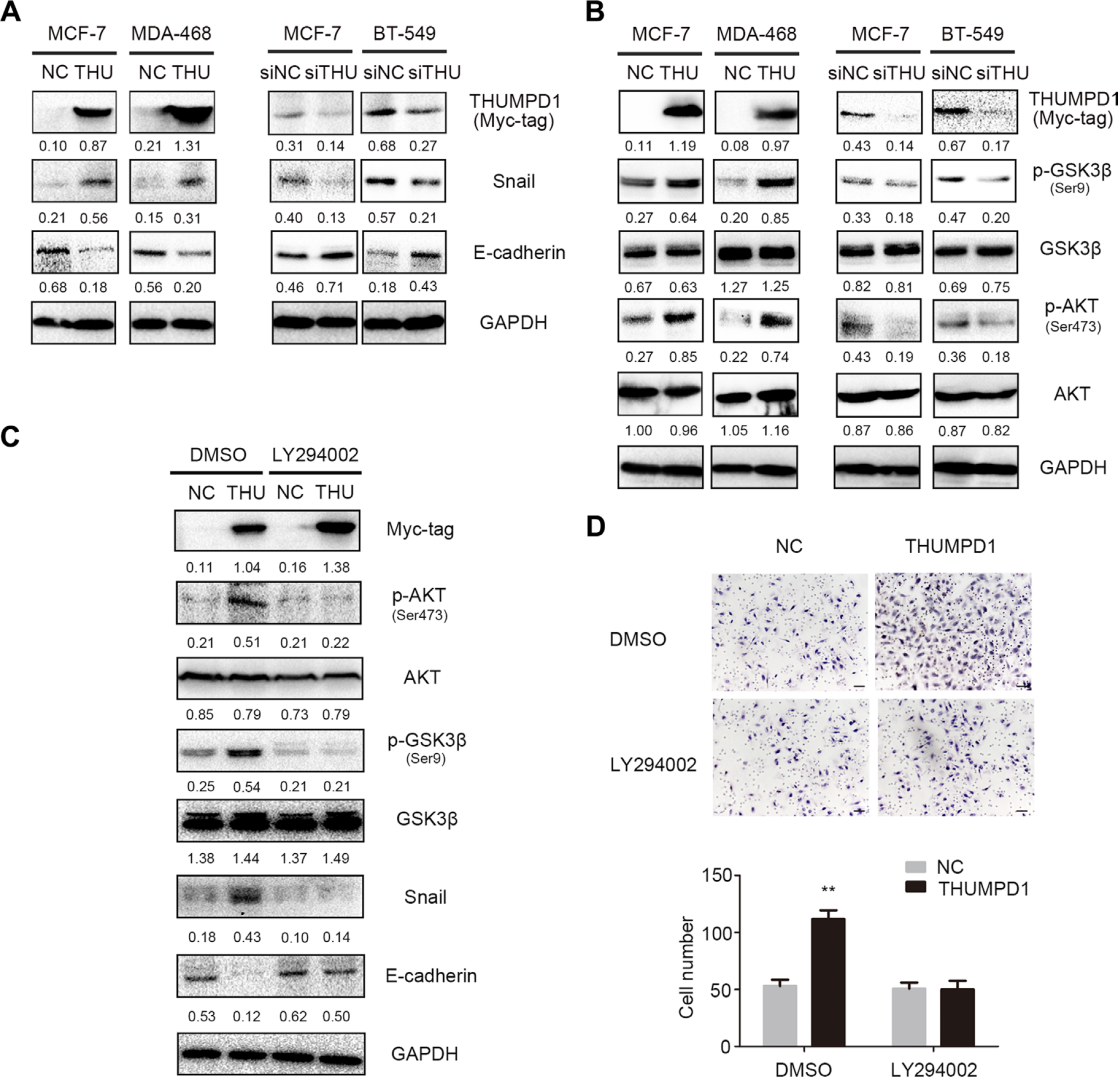
THUMPD1 promoted cancer cell invasion and migration via the AKT-GSK3β-Snail pathway THUMPD1 overexpression in MCF-7 and MDA-MB-468 cells upregulated Snail and downregulated E-cadherin, whereas THUMPD1 knockdown downregulated Snail and upregulated E-cadherin (**A**) p-AKT and its downstream target, p-GSK3β, were increased in THUMPD1-overexpressing MCF-7 and MDA-MD-468 cells, but decreased in THUMPD1-deficient MCF-7 and BT-549 cells (**B**) AKT inhibitor, LY294002 (10 μM), reversed the effects of THUMPD1 on p-AKT and p-GSK3β expression, decreasing Snail and increasing E-cadherin (**C**) and reducing THUMPD1-overexpressing MCF-7 cell invasion (**D**).

We analyzed expression of proteins involved in breast cancer cell epithelial-mesenchymal transition (EMT), with or without THUMPD1 overexpression or knockdown. Western blotting results revealed that THUMPD1 overexpression in MCF-7 and MDA-MB-468 cells upregulated Snail and downregulated E-cadherin (Figure 5A).THUMPD1 knockdown in MCF-7 and BT-549 cells downregulated Snail and upregulated E-cadherin. Levels of other proteins like Slug, P120-catenin, β-catenin, α-catenin, Occludin, Zo-1, N-cadherin, and Vimentin showed no obvious changes (Supplementary Figure 1A). THUMPD1-overexpressing cells treated with the proteasome inhibitor, MG132, showed enhanced Snail upregulation and E-cadherin downregulation (Supplementary Figure 1B), indicating that THUMPD1 may stabilize Snail protein levels.

We next examined whether THUMPD1 activated the key signaling pathway involved in Snail stabilization. THUMPD1 overexpression induced, and THUMPD1 knockdown reduced, p-AKT and its downstream target, p-GSK3β (Figure 5B).The other key signaling proteins such as Active-β-catenin, p-P65, P65, p-ERK, ERK, p-JNK, JNK, p-P38, P38, p-FAK, and FAK showed no obvious changes (Supplementary Figure 1C). At the same time, AKT inhibition by LY294002 downregulated p-GSK3β and Snail, and upregulated E-cadherin expression (Figure 5C), indicating that THUMPD1 functional activity depends on PI3K/AKT/GSK3β signaling. Accordingly, breast cancer cell invasiveness was reduced following AKT inhibition (Figure 5D).

## DISCUSSION

The present study showed that THUMPD1 is weakly expressed in normal breast cell nuclei, and strongly expressed in IDC cell nuclei. THUMPD1 is also expressed in the cytoplasm of IDC cells, and cytosolic THUMPD1 expression positively correlated with high TNM stage, lymph node metastasis, and poor patient prognosis. In cultured breast cancer cells, endogenous THUMPD1 also localized to both the cytoplasm and nucleus. Interestingly, exogenous THUMPD1 was found in the cytoplasm rather than the nucleus (Figures 1I and 3D), although the reasons for this are unclear, and must be addressed in future studies. We also examined THUMPD1 expression in both triple-negative and non-triple-negative breast cancers, and found no correlation between THUMPD1 distribution or expression and breast cancer type.

A previous study suggested that THUMPD1 might interact with the transcriptional regulator, YAP, a potential oncogene and a member of the Hippo signaling pathway [9]. Consistent with this, our results showed that THUMPD1 interacted and co-localized with YAP in both the cytoplasm and nucleus. However, changes in THUMPD1 expression had no obvious effects on Hippo pathway activity or YAP subcellular distribution. Nucleocytoplasmic shuttling of YAP may be responsible for THUMPD1 translocation; however, this hypothesis needs further investigation [10].

Our *in vivo* and *in vitro* experiments indicated that THUMPD1 enhanced breast cancer cells invasion and migration. THUMPD1 overexpression upregulated Snail and downregulated E-cadherin, suggesting that THUMPD1 may facilitate cancer invasion through Snail, which is an extremely unstable protein [14–16]. MG132, a proteasome inhibitor, further enhanced Snail expression, suggesting that THUMPD1 may stabilize Snail to promote breast cancer cells invasion. Previous studies demonstrated that the Wnt, transforming growth factor β (TGFβ), mitogen-activated protein kinase (MAPK), and phosphatidylinositol 3-kinase (PI3K)/AKT signaling pathways regulate Snail expression [17–24]. Furthermore, glycogen synthase kinase-3 β (GSK3β) and nuclear factor (NF)-κB are key proteins controlling cancer metastasis [25–30]. Our results showed that THUMPD1 overexpression upregulated p-AKT and its downstream factor, p-GSK3β. Phosphorylation inactivates GSK3β, and this inactivation may stabilize Snail [31, 32]. Our results revealed that AKT signaling inhibition by LY294002 downregulated Snail and upregulated E-cadherin, confirming that THUMPD1 decreased E-cadherin expression via the AKT-GSK3β-Snail pathway.

Sharma, *et al*. demonstrated that THUMPD1 binds NAT10, which is a biomarker in several cancer types [8]. Ma, *et al*. indicated that NAT10 upregulation promotes hepatocellular carcinoma invasion by decreasing E-cadherin [34]. Zhang, *et al*. also confirmed that NAT10 enhanced colorectal cancer invasion and correlated with poor prognosis. We speculate that THUMPD1-NAT10 binding could accelerate cancer cell invasion cooperatively. Alternatively, THUMPD1 may promote tumor invasion as a NAT10 downstream factor.

In conclusion, we observed THUMPD1 overexpression in the cytoplasm of breast cancer cells, which positively correlated with high TNM stage, lymph node metastasis, and poor patient prognosis. Although THUMPD1 interacted with YAP, no effects on other Hippo signaling pathway members were identified. THUMPD1 promoted breast cancer cells invasion and migration, and downregulated E-cadherin via the AKT-GSK3β-Snail pathway, illuminating a possible mechanism underlying THUMPD1 in breast cancer progression.

## MATERIALS AND METHODS

### Patients and clinical specimens

The study protocol was approved by the institutional review board of China Medical University. All participants provided written informed consent, and the study was conducted according to the principles expressed in the Declaration of Helsinki. Primary tumor specimens were obtained from 146 breast cancer patients, including 82 triple-negative (deficient in estrogen receptor, progesterone receptor, and Her2/neu expression) and 64 non-triple-negative tumors. All patients diagnosed with invasive ductal carcinoma (IDC) underwent complete surgical resection at the Affiliated Cancer Hospital of China Medical University between 2001 and 2003. Among the 146 IDC specimens, there were 43 cases of ductal carcinoma *in situ* (DCIS). Complete follow-up data were available for all 146 analyzed cases. Patient survival was defined as the time from the day of surgery to the end of the follow-up period or the day of death due to recurrence or metastasis. None of the patients had received radiotherapy or chemotherapy before undergoing surgical resection, and all patients were treated with routine chemotherapy after surgery.

### Cell lines

MCF-10A, MCF-7, BT-549, MDA-MB-231, and MDA-MB-468 cell lines were obtained from Shanghai Cell Bank (Shanghai, China). All cells were cultured in RPMI 1640 medium (Invitrogen, Waltham, MA, USA) containing 10% fetal calf serum (Invitrogen) and 100 IU/mL penicillin plus 100 μg/mL streptomycin (Sigma-Aldrich, St. Louis, MO, USA). Cells were grown in sterile culture dishes at 37°C in a 5% CO_2_ atmosphere, and subcultured every two days using 0.25% trypsin (Invitrogen) for cell detachment.

### Immunohistochemistry

All tissue specimens were fixed in neutral formaldehyde, embedded in paraffin, and sectioned (thickness, 4 μm). The streptavidin-peroxidase immunohistochemical method was used to improve staining. Tissue sections were incubated at 4°C overnight with THUMPD1 mouse monoclonal antibody (1:50 dilution; Santa Cruz Biotechnology, Inc., Dallas, TX, USA); phosphate-buffered saline was used as a blank control. Sections were then incubated with biotin-labeled secondary antibodies (Ultrasensitive; MaiXin, Fuzhou, China) at 37°C for 30 min, followed by diaminobenzidine for coloration.

THUMPD1 staining intensity was scored as follows: 0, none; 1, weak (staining in 1–25% of cells); 2, moderate (26–50%); 3, high (51–75% of), and 4, very high (76–100%). Individual tumor sample scores were multiplied to yield a final score of 0–12. THUMPD1 cytosolic expression was considered high in tumors with final scores ≥ 4 and negative or weak in those with final scores < 4. THUMPD1 nuclear localization was assessed in tumors with nuclear staining scores > 2. In cases where THUMPD1 localized to either the cytoplasm or nucleus, tumors were considered THUMPD1-overexpressing.

### Western blotting and immunoprecipitation

Western blotting and immunoprecipitation assays were performed as described previously [33]. Primary antibodies against THUMPD1 and LaminB1 (1:100) were purchased from Santa Cruz Biotechnology, Inc., and antibodies against GAPDH (1:500 and 1:5,000) were from Sigma (St. Louis, MO, USA). PI3 kinase inhibitor LY294002 (10 μM) and antibodies against Snail, Slug, YAP, p-YAP-Ser127, LATS, p-LATS (Ser1079), Myc-tag, Vimentin, P38, p-P38, ERK, p-ERK, JNK, p-JNK, GSK3β, p-GSK3β (Ser9), p-P65 (Ser536), AKT, p-AKT, and active β-catenin (1:1,000) were purchased from Cell Signaling Technology (Danvers, MA, USA). Antibodies against β-catenin, E-cadherin, N-cadherin, P65, and P120 catenin (1:1,000) were purchased from BD Transduction Laboratories (Lexington, KY, USA). Antibodies against Zo-1 and Occludin (1:500) were purchased from Proteintech (Chicago, IL, USA). Proteasome inhibitor MG132 (10μM) and α-tublin antibody (1:500) were purchased from Beyotime (Jiangsu, China).

### Plasmid construction and cell transfection

The pCMV6-DDK-Myc empty vector and the pCMV6-DDK-Myc-THUMPD1 vector were purchased from OriGene (Rockville, MD, USA). THUMPD1-siRNA (sc-93083) and control siRNA (sc-37007) were purchased from Santa Cruz Biotechnology. Cells were transfected using the Lipofectamine 3000 kit (Invitrogen) according to the manufacturer's instructions.

### Immunofluorescence staining

Cells were fixed with 4% paraformaldehyde, blocked with 1% bovine serum albumin, and incubated overnight with THUMPD1 monoclonal antibodies (1:100; Santa Cruz) at 4°C. Cells were then incubated with tetramethylrhodamine isothiocyanate-conjugated secondary antibodies (Cell Signaling Technology) at 37°C for 2 h. Cell nuclei were counterstained with 4′, 6-diamidino-2-phenylindole (DAPI). Epifluorescence microscopy was performed using an inverted Nikon TE300 microscope (Nikon Co., Ltd., Tokyo, Japan), and confocal microscopy was performed using a Radiance 2000 laser scanning confocal microscope (Carl Zeiss, Oberkochen, Germany).

### Matrigel invasion assay

Cell invasion assays were performed using 24-well Transwell chambers with 8-μm pores (Costar, Cambridge, MA, USA). Inserts were coated with 20μl Matrigel (1:3 dilution; BD Bioscience, San Jose, CA, USA). Cells were trypsinized 48 h after transfection, resuspended at 3 × 10^5^ cells in 100 μL of serum-free medium, and transferred to the upper chambers of Transwell plates. Fetal bovine serum (10%) was added to the lower chambers as chemoattractant. After 18 h incubation, cells that passed through the filter were fixed with 4% paraformaldehyde, stained with hematoxylin, and counted under a microscope in 10 randomly selected fields at ×40 magnification.

### Wound healing assay

Wounds were created in cell monolayers at < 90% confluence 48 h after transfection, using a 200-μl pipette tip. Cell migration into the wound was observed at different time points. Wound areas were measured using Image J software, and representative images were taken. Each experimental condition was analyzed in duplicate, and three independent experiments were performed.

### Transplantation of tumor cells into nude mice

Animals used in this study were treated according to the National Institutes of Health guide for the care and use of laboratory animals (NIH Publications No. 8023, revised 1978). Four-week-old female BALB/c nude mice were purchased from Slac (Shanghai, China) and kept in a laminar-flow cabinet under specific pathogen-free conditions for two weeks before use. Each mouse was then inoculated intravenously (tail vein) with 2 × 10^6^ THUMPD1-transfected MCF-7 tumor cells in 0.2 mL sterile PBS. Six weeks after inoculation, mice were euthanized and examined for tumor growth and dissemination. Tumors, hearts, livers, lungs, and kidneys were dissected, fixed in 4% formaldehyde (Sigma) and embedded in paraffin. Serial 6-μm-thick sections were cut, stained with hematoxylin and eosin, and examined by microscopy.

### Statistical analyses

All statistical analyses were performed using SPSS 22.0 software (SPSS Statistics, Inc., Chicago, IL, USA). Immunohistochemistry results were analyzed via chi-square and Spearman's rank correlation tests. Kaplan-Meier survival analysis results were compared using the log-rank test. The Cox regression model was used to test prognostic values. All clinicopathological parameters were included in the Cox regression model and tested by univariate and multivariate analysis according to the enter method. Differences between groups were compared using Student *t*-tests, and *p* < 0.05 was considered statistically significant.

## SUPPLEMENTARY MATERIALS FIGURES AND TABLES


