# Supplementary material for: Cytosolic THUMPD1 promotes breast cancer cells invasion and metastasis via the AKT-GSK3-Snail pathway
Source: Oncotarget. 2017 Jan 5;8(8):13357–66. doi: 10.18632/oncotarget.14528 (PMC5355103; doi:10.18632/oncotarget.14528)
Supplement: Supplementary file 1 [file oncotarget-08-13357-s001.pdf]

Cytosolic THUMPD1 promotes breast cancer cells invasion and metastasis via the AKT-GSK3-Snail pathway

Supplementary Materials

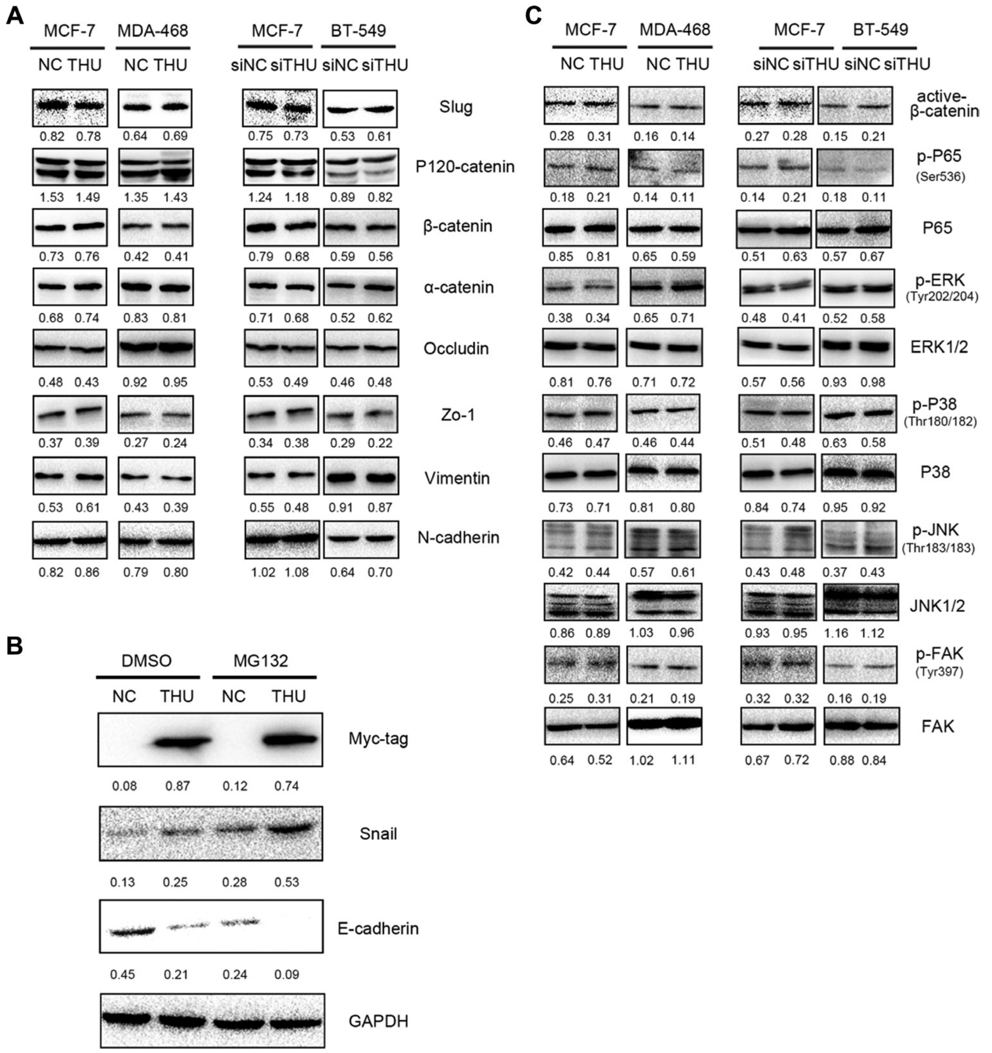

Supplementary Figure 1: Thumpd1 decreased E-cadherin via AKT-GSK3β-Snail pathway. (**A**) Overexpressing THUMPD1 did not alter the expression of Slug, P120-catenin, β-catenin, α-catenin, Occludin, Zo-1, Vimentin and N-cadherin, the levels of these proteins showed no significant changes after interfering THUMPD1 by siRNA. (**B**) When a proteasome inhibitor (10 μM MG132) is added to the culture medium, the effects of transfection on the expression of Snail are diminished or abolished, and there is a corresponding decrease in E-cadherin. (**C**). The key signaling proteins involved in stabilizing Snail had no obvious changes by regulating THUMPD1.
